# Cross-Sectional Study of Variations in Cephalometric Parameters in Arab Orthodontic Patients with Skeletal Class I and II

**DOI:** 10.3390/jcm14155292

**Published:** 2025-07-26

**Authors:** Kareem Midlej, Peter Proff, Nezar Watted, Fuad A. Iraqi

**Affiliations:** 1 Department of Clinical Microbiology and Immunology, Gray Faculty of Medicine and Health Sciences, Tel Aviv University, Tel Aviv 6997801, Israel; kareemmidlej@mail.tau.ac.il; 2 Department of Orthodontics, University Hospital of Regensburg, University of Regensburg, 93047 Regensburg, Germany; peter.proff@klinik.uni-regensburg.de; 3 Center for Dentistry Research and Aesthetics, Jatt 4491800, Israel; nezar.watted@gmx.net

**Keywords:** skeletal malocclusion, classes I and II, Arab orthodontic patients, correlation analysis, principal component analysis

## Abstract

**Objectives:** Previous literature has already discussed the effects of age and sex on the diagnosis and treatment of malocclusion problems. However, this effect varies among different ethnic groups. These differences have not yet been investigated in many populations, such as Arab orthodontic patients and residents of Israel. Therefore, it is crucial to understand such variations in specific populations for better diagnosis and treatment. The main aim of this study is to provide novel knowledge concerning skeletal classes I and II among a cohort of Arab patients who are citizens of Israel. We used parameters obtained from lateral cephalograms to understand the variations among different sex and age subgroups. We also examined the correlations and performed principal component analysis (PCA). **Methods:** This study was based on the coded records of 394 Arab patients diagnosed with skeletal Class I occlusion (SCIO) or skeletal Class II malocclusion (SCIIMO), according to the individualized ANB (Calculated_ANB) of Panagiotidis and Witt. **Results:** Among patients with SCIO, males had a significantly more horizontal growth pattern (PFH/AFH) and anterior mandible rotation (ML-NSL) than females. Regarding patients with SCIIMO, female adults had more hyperdivergent jaw bases than adolescents (ML-NL) and a more posteriorly rotated mandible (ML-NSL). Spearman’s analysis revealed many significant correlations, like Calculated_ANB, ANB angle, and Wits appraisal. The PCA results showed a remarkable ability to explain 88.6% of the sample variance using four principal components. **Conclusions:** This research revealed new information regarding Arab orthodontic patients diagnosed with skeletal class I or II. The results demonstrate the differences between the two classes. In addition, this study demonstrated the variation and correlation of cephalometric parameters among different sex and age subgroups in skeletal class I and II Arab patients, especially considering Calculated_ANB. Therefore, this study highlights the need to consider these differences when diagnosing patients and to distinguish the differences across different sex and age subgroups in the diagnosis and treatment process. Furthermore, the PCA results showed the importance of ML-NSL, SN-Pg, PFH/AFH ratio, and NL-ML in explaining the data variance.

## 1. Introduction

Orthodontic problems can influence many oral functions, such as swallowing disorders and speech sound production (phonetics) errors. These malfunctions were described by Gonçalves et al. [1] and Leavy et al. [2]. A study by Claudino and Traebert [3] found that aesthetic facial changes can affect a person’s quality of life and well-being, especially in young people. A separate study by Javidi et al. [4] showed that orthodontic treatment improved emotional and social well-being during childhood and adolescence. Malocclusion is caused by functional, dental, skeletal, or combined conditions, as described by Ardani et al. [5]. In skeletal class I (Class I occlusion: (SCIO)), individuals present a harmonious relationship between the maxilla and mandible in the sagittal direction. This is considered the ideal (skeletal) occlusion and, hence, a treatment goal for the sagittal anomalies discussed by Ardani et al. [5] and Lone et al. [6]. Furthermore, SCIO is the most frequent skeletal class worldwide and accounts for 93% of specific populations, according to a review by Lone et al. [6]. Skeletal class II malocclusion (SCIIMO) accounts for over one-third of all malocclusions worldwide and is more frequent in Caucasians, according to a recent review by Lone et al. [7].

The etiology of skeletal malocclusion is multifactorial and affected by both genetic and environmental factors, as thoroughly discussed by Cenzato et al. [8]. Inherited factors may also be involved, as described by Ghodasra and Brizuela [9,10]. Genetic studies have found that the genes MSX1, PAX9, AXIN2, ESRRB, FGF3, FGF4, FGF9, GREM2, IRF6, JAG1, LHX8, and TWIST1 are associated with an increased risk of SCIO [6], and that the genes FGFR2, MSX1, MATN1, MYOH1, ACTN3, GHR, KAT6B, HDAC4, and AJUBA are correlated with SCIIMO [11].

In 1899, Angle established his classification of occlusions based on the relationship between the buccal groove of the mandibular first permanent molar and the mesiobuccal cusp of the maxillary first permanent molar, as described by Lone et al. [6,9].

SCIIMO is a heterogeneous condition characterized by a retrognathic mandible, prognathic maxilla, or both [7]. Steiner [12] defined SCIO as an ANB angle with values ranging between 0° and 4° and SCIIMO as an ANB angle with values > 4° [12,13]. According to Jacobson [14], the “Wits” appraisal is intended as a diagnostic aid to be used in conjunction with other parameters. Throughout the years, many studies have presented equations and regression formulas considering the individual norms of the ANB angle and Wits appraisal [15,16,17,18,19].

Previous research has confirmed ethnic differences in the properties of malocclusion. A study conducted by Trottman and Elsbach [20] compared malocclusion in preschool black and white children and found significant racial differences in occlusal relations. In addition, the prevalence of Class I molar relationship was comparable in Black and White children, whereas the prevalence of Class II molar relationship was significantly greater in White children. Another study by Phelan et al. [21] evaluated the variation in SCIIMO between Mexican mestizos and Caucasians and found no difference in the maxillomandibular relationships. However, Mexican SCIIMO participants had more protrusion of both jaws and more extraordinary proclination of the incisors than white SCIIMO participants. Furthermore, Mexicans had smaller SN-FH angles and significantly more vertical tendencies than Caucasians. The distribution of malocclusions was also examined by Alhammadi et al. [22], who included 53 studies from several countries, and found that the prevalence of malocclusion among permanent mixed dentition varied. In addition, different countries have different prevalence rates. For instance, in Israel, the prevalence of Class III was 0.7% compared to 19.9% in China. Ethnic differences may have critical clinical manifestations in treatment decisions for these two ethnic groups.

To our knowledge, the cephalometric parameters of Palestinian Arab patients with SCIO and SCIIMO have not yet been investigated. Therefore, the main aim of this study was to examine the differences and variations in cephalometric parameters and their correlations among Palestinian Arab residents of Israel patients and to evaluate the skeletal situation concerning Angle’s classes. This population can be considered a permanent population of this area, with family histories dating back to numerous generations and high levels of consanguinity. Furthermore, we analyzed the effects of age and sex on cephalometric parameters. As a secondary outcome, we planned to perform a principal component analysis to identify the most relevant variables for diagnosing skeletal Class I/II. Finally, the main hypothesis was that both skeletal class I and II patients would demonstrate significant variations across different sex and age subgroups.

## 2. Materials and Methods

### 2.1. Ethical Statement

This research was conducted according to the current guidelines and followed the ethics and regulations of the University Hospital of Regensburg Ethics Committee (approval number 19-1596-101, dated 13 November 2019). In addition, this study complied with the STROBE protocol for observational studies. This study consisted of 394 coded records of Palestinian Arab citizens of Israel who were diagnosed with SCIO or SCIIMO. All cephalograms were imported while maintaining their resolution using the TIF format into Romexis 7 software, Planmeca, Finland. Calibration was performed prior to the analysis to ensure the accuracy of the landmarks. Cephalometric analysis was performed by a senior orthodontist (NW). All information collected was part of the standard of care by the orthodontists’ team at the Center for Dentistry Research and Aesthetics based in Jatt, Israel.

The research sample consisted of 394 patients with SCIO (n = 157, 40%) and SCIIMO (n = 237, 60%). The inclusion criteria were as follows:

1. Arab patients diagnosed with SCIO (−1 ≤ Calculated_ANB ≤ 1) or SCIIMO (Calculated_ANB > 1). Calculated_ANB was determined by the difference between the measured ANB and the individual ANB of Panagiotidis and Witt (Calculated_ANB = ANB − individual ANB) [15].

2. Available data from pre-treatment lateral cephalograms.

The mean age of SCIO patients was 18 (M = 18, SD = 6.2), with an age range of 8.2–48 years. Among patients with SCIO, females constituted more than half (n = 101, 64%). The mean age of patients with SCIIMO was 17 (M = 17, SD = 6.5), with an age range of 6.8–44, and here also, females were more than half of the patients in this class (n = 162, 68%). Table 1A,B summarize detailed information about the tested SCIO and SCIIMO patients.

### 2.2. Gender and Age Subgroups

In this study, we examined the effects of sex and covariates on the variation in cephalometric parameters among patients with SCIO and SCIIMO. We grouped the patients into three subgroups according to age, as per previous literature [23,24] and clinical recommendations: 0 ≤ age ≤ 13, 14 ≤ age ≤ 20, and age ≥ 21. In summary, we compared the following subgroups:

- Males, and Females
- Age subgroups
- Combination of gender and age subgroups

### 2.3. Cephalometric Variables

Sample size

The sample size was determined by the maximum number of Arab Orthodontic patients diagnosed with skeletal class I or II malocclusion within the enrolment period. Moreover, we calculated the estimated sample size needed to obtain a moderate correlation (*p* = 0.5) in the different subgroups of sex and age using the following formula [25]:

Total sample size = N = [(Zα + Zβ)/C]2 + 3

The standard normal deviate for α = Zα = 1.9600

The standard normal deviate for β = Zβ = 0.8416

C = 0.5 × ln[(1 + r)/(1 − r)] = 0.5493

The estimated sample size needed for moderate correlation was 29 patients (for each subgroup).

Complete information on all the parameters is presented in Supplementary Table S1, and the location of all parameters assessed is presented in Supplementary Figure S1. These cephalometric parameters were the most critical variables in the present study.

### 2.4. Data Analysis

Data analysis was performed using the R software (Version 4.5.1) platform and one-way analysis of variance (ANOVA) tests. Tukey post-hoc analysis was used to understand the differences between different subgroups of sex and age within the same and other classifications. More details regarding ANOVA and Tukey analysis can be found in the study by Nanda et al. [26].

In addition, we applied Spearman’s correlation and visualized the results as a heatmap correlation matrix for a better understanding of the correlations between the different cephalometric parameters among the different subgroups. Finally, to better estimate our data structure and the contribution of each variable to the sample variance, Principal Component Analysis (PCA) was performed as described by Korenius et al. [27]. Prior to performing the PCA, we examined the Kaiser-Meyer-Olkin (KMO) statistic to determine whether the data were suitable for PCA. The results showed an overall Measure of Sampling Adequacy (MSA) of 0.5. In order to increase the overall MSA, we removed the ANB_ind_ column from the data, as it was redundant with the Calculated_ANB (ANB − ANB_ind_), and the repeated MSA was equal to 0.63, which is suitable for PCA analysis. We used different figures to illustrate the importance and weight of each cephalometric parameter when calculating the principal component. In this study, we analyzed the first four components, which explained about 88.6% of the variation in our data. The level of significance was set at *p* < 0.05.

## 3. Results

### 3.1. Variations in Cephalogram Parameters

Our observations revealed many variations in cephalometric parameters in different sex and age subgroups within the same classification or when comparing SCIO with SCIIMO. We performed Tukey multiple group comparisons to detect these variations and looked for significant (*p* < 0.05) differences, which are presented in Table 2A–C and Supplementary Table S2.

### 3.2. Variations in Cephalometric Parameters Within the Same Classification

#### 3.2.1. Class I Occlusion

When comparing SCIO patients, the results showed differences in the parameters NL-ML angle, PFH/AFH ratio, ANB_ind_, SN-Ba, S-N (mm), Go Me (mm), Wits appraisal, and ML-NSL angles, as presented in Table 2A. According to our results, male patients had a significantly (*p* < 0.05) more horizontal growth pattern (PFH/AFH) and anterior rotation of the mandible (ML-NSL) than female patients. Furthermore, in males, the mandible was significantly longer (Go-Me), even though a higher Wits appraisal indicated a more distobasal jaw relationship (*p* < 0.05). Among adolescents (age 14–20), male patients presented a more mesocephalic pattern (SN-Ba) than females.

#### 3.2.2. Class II Malocclusion

Our analysis also revealed several significant differences when comparing different sex and age groups within the SCIIMO patients. In females, adults (> 21 years) had more hyperdivergent jaw bases than adolescents (ML-NL) and a more posteriorly rotated mandible (ML-NSL) (*p* < 0.05). Furthermore, adolescents presented a more horizontal growth pattern (PFH/AFH, Gonion angle) than children (age < 13 years) and adults (Gonion angle only) (*p* < 0.05). In line with this observation, adults had a more vertical growth pattern than younger patients according to the facial axis (*p* < 0.05). Finally, female children had a greater proclination of the lower incisors (−1/ML) than female adolescents (*p* < 0.05). The detailed results are presented in Table 2B.

### 3.3. Calculated_ANB (i.e., ANB-ANB Individual)

The results demonstrated a significant difference (*p* < 0.001) in the Calculated_ANB values between SCIO and SCIIMO, as well as between sub-groups of sex and age. When comparing skeletal class I and II patients, the former had a lower Calculated_ANB (M = −0.26, SD = 0.59) than the latter (M = 2, SD = 0.9) (Table 1A,B). In addition, when comparing subgroups of sex and age, SCIIMO presented significantly higher (*p* < 0.01) values of Calculated_ANB, i.e., a more pronounced skeletal class II, compared to the same or different SCIO subgroups, as shown in Supplementary Figure S2 and Table S2C.

### 3.4. Variations in Cephalometric Parameters Between Different Skeletal Classes

This study showed many differences in the cephalometric parameters when comparing different subgroups of sex and age between SCIO and SCIIMO, as presented in Supplementary Table S2.

### 3.5. Heatmap Spearman Correlations: Calculated_ANB vs. Other Cephalometric Parameters

Both SCIO and SCIIMO demonstrated many correlations between the Calculated_ANB and other parameters. Among SCIO patients, Calculated_ANB was moderately significantly correlated with the inclination of the maxilla (NL-NSL) (ρ = 0.336, *p* < 0.01), degree of prognathism of the mandible (SNB) (ρ = −0.364, *p* < 0.01), sagittal position of the chin (SN-Pg) (ρ = −0.395, *p* < 0.01), and inclination of the lower front teeth (−1/ML) (ρ = 0.377, *p* < 0.01). Moreover, Calculated_ANB showed a significantly weak correlation with the divergence of the jaw bases (NL-ML), growth pattern (gonial angle, facial axis, SN-Ba), degree of prognathism of the maxilla (SNA), parameters related to skeletal class (ANB_ind_, Wits appraisal), length of the anterior cranial base (S-N), and inclination of the upper (+1/SNL angle) and lower front teeth (−1/NB).

**Figure 1:**
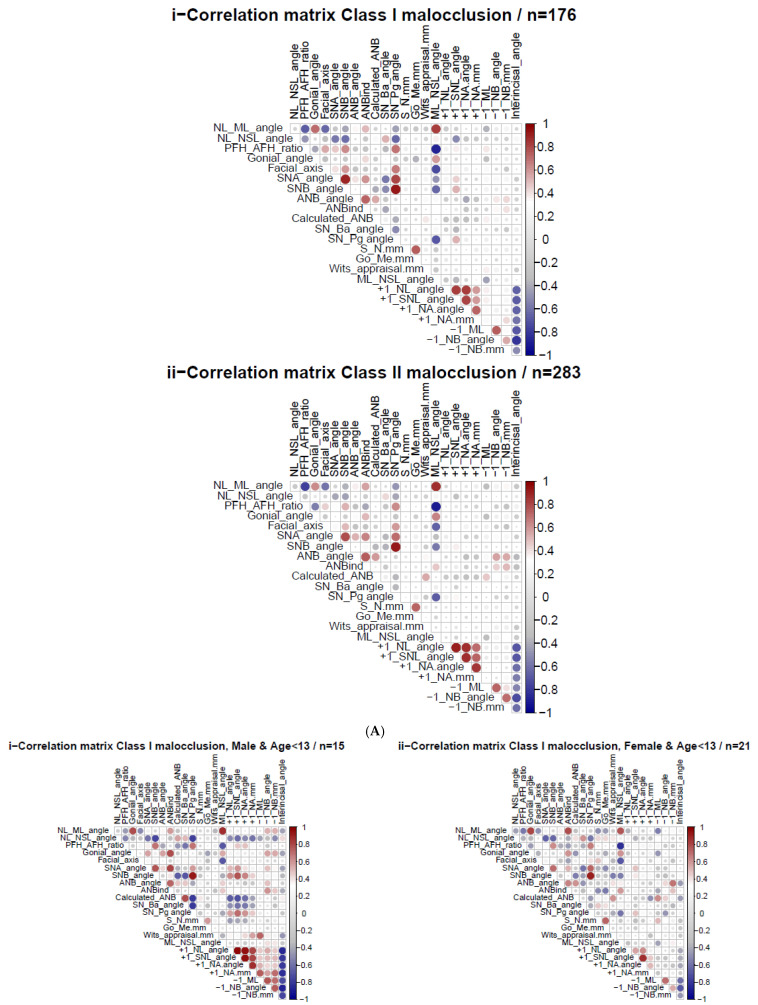

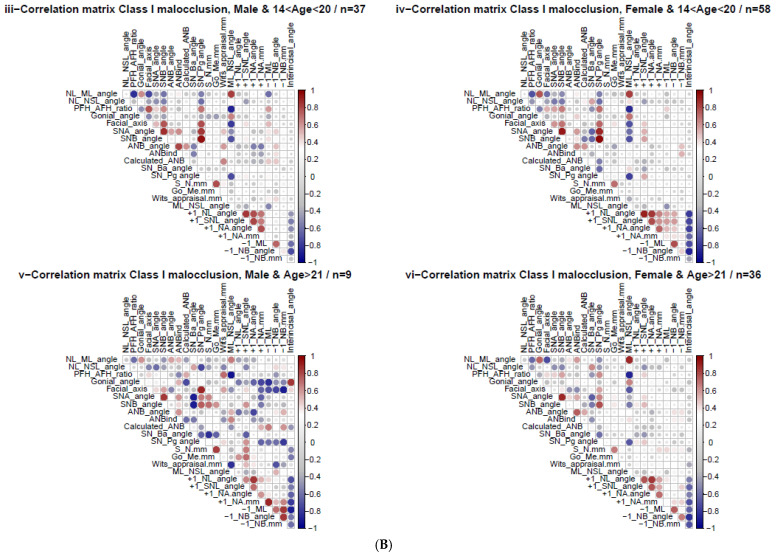

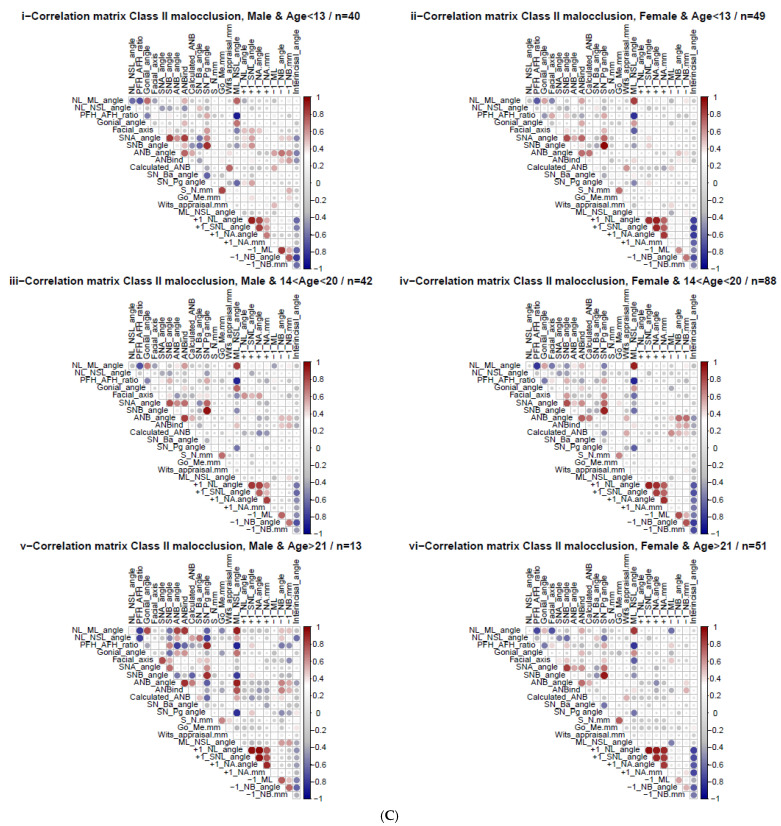
The heatmaps present the Spearman correlation between different cephalometric parameters in patients with CIO and SCIIMO. Color coding signifies the strength and direction of the correlation: blue indicates a negative correlation (strongest at ρ = −1), red indicates a positive correlation (strongest at ρ = 1), and the intensity of the color reflects the strength of the correlation. (**Ai**–**Aii**) show CIO and SCIIMO correlations regardless of gender and age. (**Bi**–**Bvi**) and (**Ci**–**Cvi**) present correlations for CIO and SCIIMO patients, respectively, further differentiated by sex and age groups.

Among these correlations, SCIIMO parameters and Calculated_ANB were: ANB angle (ρ = 0.430, *p* < 0.01), Wits appraisal (ρ = 0.574, *p* < 0.01), SN-Pg angle (ρ = −0.302, *p* < 0.01), and −1/ML (ρ = 0.334, *p* < 0.01), which were significantly weakly correlated with SNB, SN-Ba, +1/NL, +1/SNL, +1/NA angle, +1/NA (mm), −1/NB angle, and −1/NB (mm) (Table 3A and Figure 1A).

### 3.6. Heatmaps Spearman Correlations—Gender and Age Variation

The heatmaps of each sex and age subgroup revealed many significant Spearman correlations, especially between calculated_ANB and other cephalometric parameters. Among SCIO female children, the results showed a strong, significant (*p* < 0.05) correlation between Calculated_ANB on the one hand and the divergence of the jaw bases (NL-ML), growth pattern (gonial angle), ANB, and inclination of the lower incisors (−1/ML). In addition, females older than 13 years presented a significant (*p* < 0.05) correlation between Calculated_ANB and the angle of inclination of the upper jaw (NL-NSL), sagittal position of the lower jaw (SNB) and chin (SN-Pg), craniofacial pattern (SN-Ba), and inclination of the upper front teeth (+1/SNL). Regarding SCIO male patients, the results showed that patients aged 0–13 years revealed a significant (*p* < 0.05) correlation between Calculated_ANB and the sagittal location of the mandible (SNB) and chin (SN-Pg), and the inclination of the upper incisors (+1/SNL). Among males aged older than 14 years, the results revealed a significant (*p* < 0.05) correlation between Calculated_ANB and Wits appraisal, the inclination of the lower incisors (−1/ML, −1/NB), and the interincisal angle, as presented in Figure 1B and Table 3B.

Regarding SCIIMO subgroup correlations, our analysis showed that the Calculated_ANB was associated with the measured ANB angle in all subgroups except for males aged 0–13 years (*p* < 0.05). In addition, a strong correlation was observed between the Calculated_ANB and Wits appraisal in all class II subgroups (*p* < 0.05), as presented in Figure 1C and Table 3C.

### 3.7. Principal Component Analysis (PCA)

In the next stage, we performed PCA to understand how cephalometric parameters contributed to sample variability. The results showed that the first four components explained 88.6% of the variance in the data, and this is presented in Table 4A.

To evaluate the weight of each cephalometric parameter among the first four components, we extracted a loading matrix. The first component revealed a high positive value for the divergence of the jaw bases (NL-ML) and the inclination of the mandible (ML-NSL), and a high negative value for the growth pattern (PFH/AFH, facial axis) and the sagittal position of the chin (SN-Pg). The second component showed a positive value for the upper incisor inclination (+1/NL, +1/SNL, +1/NA) and position (+1/NA (mm)) and a high negative value for the interincisal angle. The third component presented a high negative value in the sagittal parameters (ANB, Calculated_ANB, and Wits appraisal) and dental parameters (−1/ML and −1/NB). Finally, the fourth component showed high negative values for the NL-NSL and SN-Ba angles and a high positive value for the SNB angle. The detailed information is presented in Table 4B.

**Figure 2:**
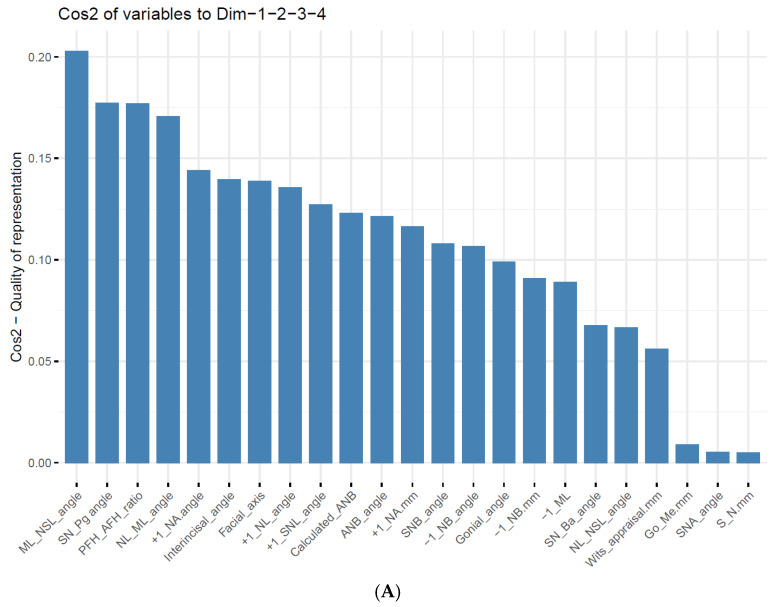

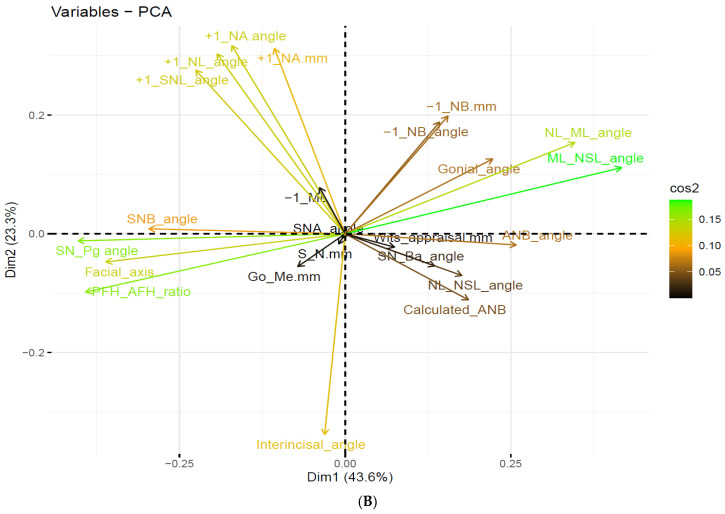
The results of a Principal Component Analysis (PCA) for diagnosing skeletal class I/II. (**A**) shows the contribution of each cephalometric parameter to the first four principal components (PCs) through their cosine squared values. The X-axis lists all variables, and the Y-axis shows the values of the Cos2 quality of presentation. (**B**) PCA biplot visualizing the relationships between the variables and the first two PCs (PC1 and PC2). The X- and Y-axes represent PC1 and PC2, respectively. Highly contributing variables (identified in Figure 2A) are colored green, while variables with lower contributions are shown in black.

Finally, we evaluated the contribution of the cephalometric parameters to the first four components using a cosine squared function. The results in Figure 2A,B revealed that the mandible’s rotation (ML-NSL), sagittal position of the chin (SN-Pg), growth pattern (PFH/AFH ratio), and divergence of the jaw bases (NL-ML) were the most critical parameters, contributing to the first four PCs.

## 4. Discussion

Our study aimed to reveal novel information about the Palestinian Arab ethnic minority who are citizens of Israel. Specifically, we focused on the differences in cephalometric parameters between various sex and age groups. To understand the effects of sex and age on cephalometric parameters, we performed multiple comparison examinations within the same class and between SCIO and SCIIMO. We also examined the correlation between the different cephalometric parameters in the overall sample, and then with sex and age effects. Moreover, we investigated the contribution of each parameter to the variation in the data using PCA.

### 4.1. Different Groups Comparisons

Among SCIO patients, male patients had a significantly (*p* < 0.05) more horizontal growth pattern (PFH/AFH), an anteriorly rotated mandible (ML-NSL), longer mandible (Go-Me), and greater sagittal distance between the jaw bases according to Wits appraisal than females (I). These results are supported by the research by Salama and Abuaffan [28], who examined lateral cephalograms of Sudanese university students with SCIO and found statistically significant differences between both sexes, especially in skeletal variables. The SNA°, SNB°, and SNPg° angles were significantly higher (*p* < 0.01) in males than in females. In addition, they found that males showed more anteriorly inclined maxillae and mandibles (NL-NSL and ML-NSL) than females (*p* < 0.01). A survey by Drevensek et al. [29] revealed that the values of PFH and AFH differed significantly between the sexes (*p* = 0.001). The means for PFH and AFH in the early mixed dentition period were 72.3 mm and 112.0 mm for boys and 66.9 mm and 106.3 mm for girls, respectively. However, our results are not aligned with those of Deshmukh et al. [30], who found no sex differences in cephalometric variables.

Among the patients with SCIIMO, significant differences were found between the different sex and age subgroups. Adults, in particular females, had more hyper-divergent jaw bases (NL-ML) and posteriorly rotated mandibles (ML-NSL) (*p* < 0.01) than adolescents. In addition, our results showed that compared with children and adolescents, adults had a more vertical growth pattern according to the facial axis. This result is partially consistent with that of Pancherz et al. [31], who found that anterior rotation of the mandible was more frequently observed in younger than in older patients. In the same study, the authors did not find significant age-dependent differences in the divergence of the jaw bases (NL-ML) among patients with SCIIMO divisions 1 or 2. Furthermore, female children showed more vertical growth patterns (PFH/AFH ratio and Gonion angle) and proclined lower incisors than female adolescents. These results are supported by Yoon and Chung [32], who found that with growth, the face becomes flatter, and the mandible rotates forward (ANB, MP-SN, and gonial angles decrease, and PFH: AFH (%) increases).

### 4.2. Calculated_ANB Correlation with Other Cephalometric Parameters

Overall, both the SCIO and SCIIMO groups demonstrated many correlations between Calculated_ANB and other parameters. Among SCIO patients, Calculated_ANB had a moderate significant association with the inclination of the upper jaw (NL-NSL) (ρ = 0.336, *p* < 0.01), the sagittal position of the mandible (SNB) (ρ = −0.364, *p* < 0.01), chin SN-Pg (ρ = −0.395, *p* < 0.01), and the lower incisors’ inclination (−1/ML) (ρ = 0.377, *p* < 0.01). In SCIIMO, Calculated_ANB was associated with ANB (ρ = 0.430, *p* < 0.01), Wits appraisal (ρ = 0.574, *p* < 0.01), the chin’s antero-posterior location (SN-Pg) (ρ = −0.302, *p* < 0.01), and the lower incisors’ inclination (−1/ML) (ρ = 0.334, *p* < 0.01). These findings demonstrate that the skeletal class, determined by Calculated_ANB, is not only correlated with the sagittal position of the jaw bases but also with other skeletal (and dental) parameters.

Our results are consistent with those of previous studies. According to Jan et al. [33], the correlation between the ANB angle and Wits was significantly correlated with an “r” value of 0.469 (r = 0.469, *p* < 0.00). Another study conducted on 60 subjects found a statistically significant correlation between the ANB angle and Wits appraisal values. In addition, Saad et al. [34] found a statistically insignificant correlation between the SNP plane angle and ANB angle and Wits appraisal. In another study performed by Ardani et al. [35] on SCIIMO in the Japanese population, a significant correlation was found between mandibular length and other skeletal variables in the vertical and sagittal directions, such as facial axis, SN-MP, LAFH, and ANB. An investigation done by Gul-e-Erum and Fida [36] examined cephalometric analysis for assessing the sagittal jaw relationship and determining the relationship between them and found a robust correlation between AXB and AF-BF distance and a weak correlation between ANB and Beta angle. In the same study, they found that the Wits appraisal showed the highest coefficient of variability.

### 4.3. Results of Principal Component Analysis

Subsequently, after understanding the variations and correlations of the different cephalometric parameters, we aimed to better understand our data structure and the contribution of each parameter to this variation. We conducted a PCA analysis that showed us the ability of the first four components to explain 88.6% of the variation in the skeletal class diagnosis. The results revealed that the inclination of the lower jaw (ML-NSL), sagittal position of the chin (SN-Pg), growth pattern (PFH/AFH ratio), and divergence of the jaw bases (NL-ML) were the most important parameters for the first four PCs. In a previous study by Jan et al. [33] that examined sixty-three lateral cephalometric variables of 309 white Class II adults, PCA resulted in seven principal components that explained 81% of the variation. The first three components represented variations in mandibular rotation, maxillary incisor angulation, and mandibular length. Another study by Moreno et al. [37], which analyzed the phenotype–genotype correlations of facial width and height proportions in patients with Class II malocclusion using 2D frontal repose photographs, revealed that four principal components (PCs) explained 75% of the total variation. Furthermore, Dascalu and Zegan [38] performed a previous study that used 16 measurements and a sample of 120 patients. A cephalometric study was performed to identify possible differences between the measures for different types of orthodontic diagnoses, and five principal components were identified, which covered 88.54% of the total variance of the variables. The rotated component matrix showed that the components corresponded to the following measurement order: SND, Maxl-NA, 1I-NB S-E, and ANB.

### 4.4. Limitations

The study sample comprised only patients with skeletal classes I and II, while those with skeletal class III were excluded. Furthermore, this study was based on a single rater’s diagnosis. Another limitation was the heterogeneous size of the age- and sex-specific subgroups, which can be explained by the retrospective study of the enrollment of patients without consideration of subgroups.

## 5. Conclusions and Future Research

This research revealed new information regarding Arab orthodontic patients who are diagnosed with skeletal class I or II. The results demonstrate the differences between the two classes. In addition, this study demonstrated the variation and correlation of cephalometric parameters among different sex and age subgroups in skeletal class I and II Arab patients, especially considering Calculated_ANB. Therefore, this study highlights the need to consider these differences when diagnosing patients and to distinguish the differences across different sex and age subgroups in the diagnosis and treatment process. For example, the results showed that class I male patients had a significantly (*p* < 0.05) more horizontal growth pattern (PFH/AFH), an anteriorly rotated mandible (ML-NSL), longer mandible (Go-Me), and greater sagittal distance between the jaw bases according to Wits appraisal than females. The PCA showed that a few cephalometric parameters explained 88.6% of the total variance in skeletal class I/II diagnosis. These parameters are the inclination of the lower jaw (ML-NSL), sagittal position of the chin (SN-Pg), growth pattern (PFH/AFH ratio), and divergence of the jaw bases (NL-ML), which have a strong influence on Calculated_ANB and must be identified with high certainty to precisely define an individual’s skeletal class.

## Figures and Tables

**Table 1:** Detailed information about SCIO patients. (N)- sample size, (M)-Mean, (Std. Dev.)—Standard Deviation, (Min)—Minimum Value, (Pctl. 25)—25th percentile, (Pctl. 75)—75th percentile, (Max)-Maximum Value (1A), while Table 1B shows the information about the tested SCIIMO patients. (N)—sample size, (M)—Mean, (Std. Dev.)—Standard Deviation, (Min)—Minimum Value, (Pctl. 25)—25th percentile, (Pctl. 75)—75th percentile, (Max)—Maximum Value. (A). Skeletal class I descriptive statistics. (B). Skeletal class II descriptive statistics.

(**A**)							
	**Skeletal Class I**						
Variable	N	Mean	Std. Dev.	Min	Pctl. 25	Pctl. 75	Max
Age	157	18	6.2	8.2	14	21	48
0 < Age < 13	42(27%)						
14 < Age < 20	80 (51%)						
Age > 21	35 (22%)						
Female	101 (64%)						
Male	56 (36%)						
NL-ML angle	157	30	6.2	13	26	34	46
NL-NSL angle	157	7.7	3.3	−0.1	5.4	9.6	16
PFH/AFH	157	65	5	50	62	68	79
Gonial angle	157	132	7.6	113	128	138	155
Facial axis	157	88	4.3	77	85	91	102
SNA angle	157	83	3.8	75	80	85	94
SNB angle	157	78	3.3	68	75	80	85
ANB angle	157	5.1	1.5	2.2	4.1	6	9
ANB_ind_	157	5.4	1.5	2.2	4.3	6.3	8.8
Calculated_ANB (ANB − ANB_ind_)	157	−0.26	0.59	−1	−0.8	0.1	1
SN-Ba angle	157	129	5.7	114	125	133	142
SN-Pg angle	157	78	3.7	67	76	80	89
S-N (mm)	157	63	7.2	39	58	66	88
Go-Me (mm)	157	60	6.3	46	56	63	82
Wits appraisal (mm)	157	−3.3	2.6	−9.1	−5.3	−1.5	3
ML-NSL angle	157	37	6.9	7.5	33	41	61
+1/NL angle	157	114	5.5	97	110	118	128
+1/SNL angle	157	105	10	11	101	111	125
+1/NA angle	157	24	5.6	2.8	20	27	40
+1/NA (mm)	157	3.4	2.1	−2.3	2	5.1	8.6
−1/ML (anatomic)	157	93	6.7	77	89	98	111
−1/NB angle	157	28	6.3	8.5	24	32	47
−1/NB (mm)	157	5.6	2.4	−1.4	3.9	7.4	12
Interincisal angle	157	124	9.8	102	118	129	154
(**B**)							
	**Skeletal Class II**						
Variable	N	Mean	Std. Dev.	Min	Pctl. 25	Pctl. 75	Max
Age	237	17	6.5	6.8	13	21	44
0 < Age < 13	74 (31%)						
14 < Age < 20	107 (45%)						
Age > 21	56 (24%)						
Female	162 (68%)						
Male	75 (32%)						
NL-ML angle	237	28	6.5	13	24	33	54
NL-NSL angle	237	8.3	3.6	1	6.1	10	42
PFH/AFH	237	65	5.3	50	62	69	79
Gonial angle	237	128	7.6	110	124	134	150
Facial axis	237	88	4.6	70	85	91	102
SNA angle	237	83	3.7	75	80	86	94
SNB angle	237	75	5.4	7.8	73	78	84
ANB angle	237	7.2	1.8	3.3	6	8.3	16
ANB_ind_	237	5.3	1.6	1.6	4.1	6.4	10
Calculated_ANB (ANB − ANB_ind_)	237	2	0.9	1.1	1.3	2.6	5.5
SN-Ba angle	237	130	5.3	119	126	133	147
SN-Pg angle	237	76	3.5	65	74	79	85
S-N (mm)	237	63	6.9	30	59	65	87
Go-Me (mm)	237	59	5.9	44	55	62	77
Wits appraisal (mm)	237	−1.2	2.5	−9.5	−2.8	0.6	8.3
ML-NSL angle	237	36	7.2	4.6	32	40	63
+1/NL angle	237	113	7.6	91	108	119	135
+1/SNL angle	237	105	8.3	78	99	110	124
+1/NA angle	237	21	12	−121	17	27	42
+1/NA (mm)	237	2.9	2.3	−2.8	1.2	4.4	9.2
−1/ML (anatomic)	237	97	9.3	9.5	92	102	120
−1/NB angle	237	29	7	6.8	25	33	50
−1/NB (mm)	237	5.6	2.6	−0.5	3.8	7.4	12
Interincisal angle	237	122	11	100	114	129	163

**Table 2:** Multiple group comparisons of cephalometric parameters performed using the Tukey method. Significant differences are indicated by *p*-values less than 0.01 and 0.05. (A) compares sex and age within the CIO patient groups, and (B) compares sex and age within the same SCIIMO patient groups. Finally, (C) compares the Calculated_ANB parameter by sex and age across different classes. A—Skeletal class I multiple comparison analysis. B—Skeletal class II multiple comparison analysis. C—Calculated ANB sub-group comparisons.

(**A**)					
**Parameter**	**Group A _ Group B**	**Difference**	**Lower CI**	**Upper CI**	**Adj. *p* Value**
NL-ML angle	I_Male-I_Female	−2.11	−4.12	−0.10	0.04
PFH/AFH	I_Male-I_Female	2.56	0.34	4.77	0.02
PFH/AFH	I_Male_14 < Age < 20-I_Female_0 < Age < 13	4.03	0.26	7.80	0.03
ANB_ind_	I_Male-I_Female	−0.51	−1.00	−0.02	0.04
SN-Ba	I_Male_14 < Age < 20-I_Female_14 < Age < 20	−3.96	−7.66	−0.26	0.03
S-N (mm)	I_Male-I_Female	3.43	0.47	6.38	0.02
S-N (mm)	I_Male_14 < Age < 20-I_Female_0 < Age < 13	7.03	0.98	13.09	0.01
Go Me (mm)	I_Male-I_Female	2.16	0.12	4.20	0.04
Wits appraisal	I_Male-I_Female	1.01	0.15	1.86	0.02
ML-NSL angle	I_Male-I_Female	−3.04	−6.08	−0.01	0.05
(**B**)					
**Parameter**	**Group A _ Group B**	**Difference**	**Lower CI**	**Upper CI**	**Adj. *p* value**
NL-ML angle	II_Age > 21-II_14 < Age < 20	4.21	1.24	7.18	0.00
NL-ML angle	II_Female_Age > 21-II_Female_14 < Age < 20	4.43	0.48	8.38	0.01
PFH/AFH	II_Age > 21-II_14 < Age < 20	−2.66	−5.09	−0.24	0.02
PFH/AFH	II_14 < Age < 20-II_0 < Age < 13	1.98	0.11	3.85	0.03
PFH/AFH	II_Female_14 < Age < 20-II_Female_0 < Age < 13	3.08	0.23	5.93	0.03
PFH/AFH	II_Female_Age > 21-II_Female_14 < Age < 20	−2.95	−5.81	−0.08	0.04
Gonial angle	II_14 < Age < 20-II_0 < Age < 13	−3.01	−5.68	−0.34	0.02
Gonial angle	II_Age > 21-II_14 < Age < 20	3.37	0.46	6.28	0.02
Facial axis	II_Age > 21-II_0 < Age < 13	−2.50	−4.75	−0.24	0.02
Facial axis	II_Age > 21-II_14 < Age < 20	−2.42	−4.52	−0.32	0.01
Facial axis	II_Female_Age > 21-II_Female_14 < Age < 20	−3.00	−5.79	−0.21	0.02
Facial axis	II_Female_Age > 21-II_Female_14 < Age < 20	−3.00	−5.49	−0.50	0.01
Facial axis	II_Male_0 < Age < 13-II_Female_Age > 21	3.17	0.07	6.27	0.04
SNB angle	II_14 < Age < 20-II_0 < Age < 13	2.00	0.08	3.91	0.04
SN-Pg angle	II_14 < Age < 20-II_0 < Age < 13	1.31	0.09	2.52	0.03
SN-Pg angle	II_Age > 21-II_14 < Age < 20	−1.60	−2.93	−0.28	0.01
S-N (mm)	II_Male-II_Female	2.35	0.49	4.22	0.01
S-N (mm)	II_Male_0 < Age < 13-II_Female_14 < Age < 20	4.39	0.18	8.60	0.04
ML-NSL angle	II_Age > 21-II_14 < Age < 20	3.99	0.68	7.31	0.01
ML-NSL angle	II_Female_Age > 21-II_Female_14 < Age < 20	4.38	0.47	8.29	0.02
+1/NA angle	II_14 < Age < 20-II_0 < Age < 13	−5.29	−9.62	−0.95	0.01
+1/NA angle	II_Male_14 < Age < 20-II_Female_0 < Age < 13	−9.97	−17.57	−2.37	0.00
+1/NA angle	II_Male_14 < Age < 20-II_Male_0 < Age < 13	−9.27	−17.59	−0.96	0.01
+1/NA (mm)	II_14 < Age < 20-II_0 < Age < 13	−1.27	−2.23	−0.32	0.00
+1/NA (mm)	II_Age > 21-II_0 < Age < 13	−1.01	−1.96	−0.06	0.03
−1/ML (anatomic)	II_Female_14 < Age < 20-II_Female_0 < Age < 13	5.34	0.19	10.48	0.03
(**C**)					
**Parameter**	**Group A _ Group B**	**Difference**	**Lower CI**	**Upper CI**	**Adj. *p* value**
Calculated_ANB	II_Male-I_Female	2.24	1.93	2.55	0
Calculated_ANB	II_Female-I_Male	2.17	1.85	2.48	0
Calculated_ANB	II_Male-I_Male	2.12	1.76	2.48	0
Calculated_ANB	II_0 < Age < 13-I_0 < Age < 13	2.24	1.81	2.68	0
Calculated_ANB	II_14 < Age < 20-I_0 < Age < 13	2.18	1.76	2.59	0
Calculated_ANB	II_Age > 21-I_0 < Age < 13	2.05	1.59	2.51	0
Calculated_ANB	II_0 < Age < 13-I_14 < Age < 20	2.28	1.92	2.65	0
Calculated_ANB	II_14 < Age < 20-I_14 < Age < 20	2.22	1.88	2.55	0
Calculated_ANB	II_Age > 21-I_14 < Age < 20	2.09	1.7	2.49	0
Calculated_ANB	II_0 < Age < 13-I_Age > 21	2.42	1.95	2.88	0
Calculated_ANB	II_14 < Age < 20-I_Age > 21	2.35	1.91	2.79	0
Calculated_ANB	II_Age > 21-I_Age > 21	2.23	1.74	2.71	0
Calculated_ANB	II_Female_0 < Age < 13-I_Female_0 < Age < 13	2.2	1.55	2.85	0
Calculated_ANB	II_Female_14 < Age < 20-I_Female_0 < Age < 13	2.29	1.69	2.9	0
Calculated_ANB	II_Female_Age > 21-I_Female_0 < Age < 13	2.09	1.43	2.74	0
Calculated_ANB	II_Male_0 < Age < 13-I_Female_0 < Age < 13	2.38	1.68	3.09	0
Calculated_ANB	II_Male_14 < Age < 20-I_Female_0 < Age < 13	2.01	1.31	2.71	0
Calculated_ANB	II_Male_Age > 21-I_Female_0 < Age < 13	2.07	1.18	2.96	0
Calculated_ANB	II_Female_0 < Age < 13-I_Female_14 < Age < 20	2.27	1.73	2.81	0
Calculated_ANB	II_Female_14 < Age < 20-I_Female_14 < Age < 20	2.36	1.88	2.84	0
Calculated_ANB	II_Female_Age > 21-I_Female_14 < Age < 20	2.16	1.61	2.7	0
Calculated_ANB	II_Male_0 < Age < 13-I_Female_14 < Age < 20	2.45	1.85	3.06	0
Calculated_ANB	II_Male_14 < Age < 20-I_Female_14 < Age < 20	2.08	1.49	2.67	0
Calculated_ANB	II_Male_Age > 21-I_Female_14 < Age < 20	2.14	1.32	2.95	0
Calculated_ANB	II_Female_0 < Age < 13-I_Female_Age > 21	2.33	1.7	2.97	0
Calculated_ANB	II_Female_14 < Age < 20-I_Female_Age > 21	2.42	1.84	3.01	0
Calculated_ANB	II_Female_Age > 21-I_Female_Age > 21	2.22	1.58	2.86	0
Calculated_ANB	II_Male_0 < Age < 13-I_Female_Age > 21	2.51	1.82	3.2	0
Calculated_ANB	II_Male_14 < Age < 20-I_Female_Age > 21	2.14	1.46	2.82	0
Calculated_ANB	II_Male_Age > 21-I_Female_Age > 21	2.2	1.32	3.08	0
Calculated_ANB	II_Female_0 < Age < 13-I_Male_0 < Age < 13	2.12	1.38	2.87	0
Calculated_ANB	II_Female_14 < Age < 20-I_Male_0 < Age < 13	2.21	1.51	2.91	0
Calculated_ANB	II_Female_Age > 21-I_Male_0 < Age < 13	2.01	1.26	2.76	0
Calculated_ANB	II_Male_0 < Age < 13-I_Male_0 < Age < 13	2.3	1.51	3.09	0
Calculated_ANB	II_Male_14 < Age < 20-I_Male_0 < Age < 13	1.93	1.15	2.71	0
Calculated_ANB	II_Male_Age > 21-I_Male_0 < Age < 13	1.99	1.03	2.95	0
Calculated_ANB	II_Female_0 < Age < 13-I_Male_14 < Age < 20	2.12	1.5	2.73	0
Calculated_ANB	II_Female_14 < Age < 20-I_Male_14 < Age < 20	2.21	1.65	2.76	0
Calculated_ANB	II_Female_Age > 21-I_Male_14 < Age < 20	2	1.39	2.61	0
Calculated_ANB	II_Male_0 < Age < 13-I_Male_14 < Age < 20	2.3	1.63	2.96	0
Calculated_ANB	II_Male_14 < Age < 20-I_Male_14 < Age < 20	1.92	1.27	2.58	0
Calculated_ANB	II_Male_Age > 21-I_Male_14 < Age < 20	1.98	1.12	2.84	0
Calculated_ANB	II_Female_0 < Age < 13-I_Male_Age > 21	2.38	1.38	3.38	0
Calculated_ANB	II_Female_14 < Age < 20-I_Male_Age > 21	2.47	1.5	3.44	0
Calculated_ANB	II_Female_Age > 21-I_Male_Age > 21	2.27	1.27	3.27	0
Calculated_ANB	II_Male_0 < Age < 13-I_Male_Age > 21	2.56	1.53	3.6	0
Calculated_ANB	II_Male_14 < Age < 20-I_Male_Age > 21	2.19	1.16	3.22	0
Calculated_ANB	II_Male_Age > 21-I_Male_Age > 21	2.25	1.08	3.42	0

**Table 3:** This table presents the Spearman correlation values between the cephalometric parameters. Significant differences are indicated by *p*-values less than 0.01 (**) and 0.05 (*). Table 3A—separately presents the correlation between Calculated_ANB and other cephalometric parameters among CIO and SCIIMO. Table 3B,C present the correlations between the Calculated_ANB and other cephalometric parameters for CIO and SCIIMO patients, respectively, further differentiated by sex and age groups. (A). Skeletal class I/II Spearman correlations of the Calculated_ANB with other cephalometric parameters. (B). Skeletal class I Spearman correlations of the Calculated_ANB with the other cephalometric parameters with sex effect. (C). Skeletal Class II Spearman correlations of the Calculated_ANB with the other cephalometric parameters with sex effect.

(**A**)						
**Class**	**I**			**II**		
	Calculated_ANB			Calculated_ANB		
NL-ML angle	−0.173 *			−0.05		
NL-NSL angle	0.336 **			0.108		
PFHAFH ratio	−0.065			−0.013		
Gonial angle	−0.276 **			0.005		
Facial axis	−0.219 **			−0.035		
SNA angle	−0.282 **			−0.047		
SNB angle	−0.364 **			−0.274 **		
ANB angle	0.112			0.430 **		
ANB_ind_	−0.255 **			−0.028		
SN-Ba angle	0.162 *			0.216 **		
SN-Pg angle	−0.395 **			−0.302 **		
S-N mm	0.12			0.052		
Go-Me (mm_	0.011			−0.059		
Wits appraisal	0.177 *			0.574 **		
ML-NSL angle	0.008			0.005		
+1/NL angle	−0.155			−0.209 **		
+1/SNL angle	−0.259 **			−0.242 **		
+1/NA angle	−0.137			−0.245 **		
+1/NA (mm)	0.06			−0.151 *		
−1/ML (anatomic)	0.377 **			0.334 **		
−1/NB angle	0.253 **			0.240 **		
−1/NB (mm)	0.003			0.152 *		
Interincisal angle	−0.107			−0.047		
(**B**)						
**Class I**	**Calculated_ANB**					
	Female			Male		
	0 < Age_13	14 < Age < 20	Age > 21	0 < Age_13	14 < Age < 20	Age > 21
NL-ML angle	−0.519 **	−0.022	0.021	−0.26	−0.133	−0.443
NL-NSL angle	0.439 *	0.436 **	0.627 **	0.287	0.033	0.144
PFH/AFH	0.146	−0.195	−0.241	−0.203	0.146	0.503
Gonial angle	−0.643 **	−0.254	−0.074	−0.312	−0.139	−0.467
Facial axis	0.412 *	−0.510 **	−0.378	−0.33	−0.011	−0.012
SNA angle	−0.225	−0.426 **	−0.545 **	−0.482	0.169	−0.072
SNB angle	−0.297	−0.509 **	−0.612 **	−0.516 *	0.032	−0.072
ANB angle	−0.058	0.185	0.043	0.078	0.28	−0.422
ANB_ind_	−0.530 **	−0.179	−0.327	−0.411	0.051	−0.448
SN-Ba angle	0.299	0.289 *	0.502 **	0.009	−0.217	−0.048
SN-Pg angle	−0.314	−0.556 **	−0.537 **	−0.502 *	−0.031	−0.192
S-N (mm)	0.29	0.013	0.059	0.221	0.166	0.132
Go-Me (mm)	0.397 *	−0.075	−0.07	0.007	0.035	0.12
Wits appraisal (mm)	0.281	0.185	0.219	0.339	−0.051	-0.771 *
ML-NSL angle	−0.314	0.182	0.243	−0.027	−0.067	−0.347
+1/NL angle	0.187	−0.268	−0.269	−0.435	−0.11	0.572
+1/SNL angle	0.001	−0.435 **	−0.352	−0.483 *	−0.083	0.467
+1/NA angle	0.152	−0.175	−0.226	−0.279	−0.26	0.743 *
+1/NA (mm)	0.081	0.207	0.031	−0.171	−0.03	0.392
−1/ML (anatomic)	0.551 **	0.264	0.338	−0.182	0.514 **	0.587
−1/NB angle	0.234	0.189	0.38	−0.257	0.501 **	0.144
−1/NB (mm)	−0.193	0.166	−0.138	−0.268	0.169	−0.156
Interincisal angle	−0.242	0.009	−0.106	0.337	−0.380 *	−0.695
(**C**)						
**Class II**	**Calculated_ANB**					
	Female			Male		
	0 < Age_13	14 < Age < 20	Age > 21	0 < Age_13	14 < Age < 20	Age > 21
NL-ML angle	−0.171	0.064	−0.049	−0.191	−0.216	0.086
NL-NSL angle	0.19	0.002	0.069	0.051	0.256	0.535
PFH/AFH	0.265	−0.076	−0.012	0.107	0.033	−0.335
Gonial angle	−0.017	−0.026	−0.054	−0.13	0.05	0.247
Facial axis	0.023	−0.041	−0.075	0.021	−0.258	0.058
SNA angle	−0.044	−0.03	0.044	−0.317	−0.012	0.127
SNB angle	−0.249	−0.285 *	−0.205	−0.364 *	−0.201	−0.369
ANB angle	0.447 **	0.554 **	0.407 **	0.137	0.403 *	0.673 *
ANB_ind_	−0.115	0.054	0.067	−0.349	0.017	0.432
SN-Ba angle	0.286	0.163	0.166	0.187	0.288	0.286
SN-Pg angle	−0.222	−0.404 **	−0.247	−0.28	−0.191	−0.327
S-N (mm)	−0.035	0.041	0.338 *	0.004	−0.224	0.155
Go-Me (mm)	−0.084	−0.055	0.216	−0.183	−0.279	0.291
Wits appraisal (mm)	0.468 **	0.629 **	0.438 **	0.667 **	0.481 **	0.853 **
ML-NSL angle	−0.145	0.051	−0.004	−0.129	0.032	0.331
+1/NL angle	−0.172	−0.136	−0.456 **	−0.106	−0.441 *	−0.296
+1/SNL angle	−0.214	−0.162	−0.411 **	−0.09	−0.439 *	−0.412
+1/NA angle	−0.231	−0.147	−0.433 **	−0.024	−0.626 **	−0.449
+1/NA (mm)	−0.300 *	0.036	−0.360 *	0.15	−0.521 **	−0.37
−1/ML (anatomic)	0.394 **	0.357 **	0.117	0.502 **	0.14	0.643 *
−1/NB angle	0.207	0.331 **	−0.012	0.209	0.107	0.648 *
−1/NB (mm)	0.05	0.253 *	0.073	0.021	0.066	0.454
Interincisal angle	0.007	−0.17	0.238	−0.168	0.219	−0.452

**Table 4:** Results of Principal component analysis (PCA) performed on cephalometric variables. (A) shows the four principal component analyses (PCA 1–4) of the cephalometric variables. Columns of Comp.1, Comp.2, Comp.3, and Comp.4 show Component 1, 2, 3, and 4 analyses, respectively, with the standard deviation for every component, the proportion of variance that each component explains, and the cumulative proportion of variance. The first four components explain 88.6% of the variance. (B) presents the PCA loading matrices. Each cell reflects the contribution of a specific cephalometric parameter to a particular component (Comp. 1–4). Positive values indicate a positive association, while negative values (shown in bold) indicate a negative association between the variable and the component. (A). Principal Component Analysis Results. (B). PCA loading matrix.

(**A**)				
	**Comp.1**	**Comp.2**	**Comp.3**	**Comp.4**
Standard deviation	1.10	0.80	0.65	0.42
Proportion of Variance	0.43	0.23	0.15	0.06
Cumulative Proportion	0.43	0.66	0.82	0.886
(**B**)				
**Parameter**	**Comp.1**	**Comp.2**	**Comp.3**	**Comp.4**
NL-ML angle	0.31	0.19	0.19	0.26
NL-NSL	0.16	−0.09	0.03	−0.41
PFH/AFH	−0.36	−0.12	−0.17	0.05
Gonial angle	0.20	0.16	0.26	0.17
Facial axis	−0.33	−0.06	−0.04	−0.17
SNA angle	0.00	−0.01	−0.06	0.13
SNB angle	−0.27	0.01	0.01	0.33
ANB angle	0.23	−0.02	−0.33	0.19
Calculated_ANB	0.17	−0.14	−0.41	−0.14
SN-Ba angle	0.12	−0.07	0.06	−0.50
SN-Pg angle	−0.37	−0.01	0.02	0.28
S-N (mm)	−0.01	−0.02	−0.10	−0.03
Go-Me (mm)	−0.06	−0.07	−0.03	0.01
Wits appraisal (mm)	0.07	−0.03	−0.34	−0.06
ML-NSL angle	0.38	0.14	0.19	0.05
+1/NL angle	−0.18	0.38	0.02	−0.18
+1/SNL angle	−0.20	0.34	−0.02	−0.02
+1/NA angle	−0.16	0.39	0.11	−0.23
+1/NA (mm)	−0.10	0.39	0.09	−0.15
−1/ML (anatomic)	−0.03	0.10	−0.43	−0.10
−1/NB angle	0.13	0.23	−0.33	0.12
−1/NB (mm)	0.14	0.25	−0.22	0.20
Interincisal angle	−0.03	−0.42	0.24	0.03

## Data Availability

The original contributions presented in this study are included in the article/Supplementary Material. Further inquiries should be directed to the corresponding author.

## Supplementary Materials

The following supporting information can be downloaded at: https://www.mdpi.com/article/10.3390/jcm14155292/s1. Table S1: Relevant cephalometric variables were analyzed, including their definition and position in Figure S1 for angular measurements; Table S2: Multiple group comparisons with the Tukey method between cephalometric parameters of CIO and SCIIMO patients. Significant differences are indicated by *p*-values less than 0.01 and 0.05. Figure S1: The most crucial points extracted from the lateral cephalogram in this study (Figure S1A). These points include the nasion (N), sella (S), pterygoid point (Pt), basion (Ba), orbitale (Or), subspinale (“A” Point), Supramentale (“B” point), and other points. Figure S1B shows the tooth axis of the upper and lower incisors positions and angles; Figure S2: Calculated_ANB variation in CIO vs. SCIIMO (density plots), among the different studied groups including female_0 < Age < 13; female_14 < Age < 20; female_Age > 21; male_0 < Age < 13; male_14 < Age < 20; and male_Age > 21. The X-axis shows the Calculated_ANB values, and the Y-axis shows the density of the calculated_ANB in the population.
