# Supplementary material for: Cross-Sectional Study of Variations in Cephalometric Parameters in Arab Orthodontic Patients with Skeletal Class I and II
Source: J Clin Med. 2025 Jul 26;14(15):5292. doi: 10.3390/jcm14155292 (PMC12347226; doi:10.3390/jcm14155292)

Supplementary Table S1.

Dimension/Group	Parameter	Unit	Definition
Vertical Analysis	NL/ML (anatomic)	°	The angle between the NL and ML
Vertical Analysis	SNL/ML (anatomic)	°	The inclination of the mandible (mandibular inclination) relative to the nasion-sella line (anterior skull base, SNL)
Vertical Analysis	NL/NSL	°	The angle between Sella-Nasion-line (NSL = SN) and nasal line (Spa-Spp)
Vertical Analysis	PFH/AFH	%	The ratio between posterior (SGo) and anterior (NMe) facial height
Vertical Analysis	Gonial Angle	°	The angle between ML and line GoAr at Gonion
Vertical Analysis	Facial axis	°	The angle between the lines NBa and PtGN’
Sagittal Analysis	Angle SNA	°	The angle between Sella, Nasion, and point A
Sagittal Analysis	Angle SNB	°	The angle between Sella, Nasion, and point B
Sagittal Analysis	ANB	°	The angle between Nasion, point A, and point B
Sagittal Analysis	ANB(indl.)	°	$ANB_{ind} = (-35.16 + 0.4 \cdot SNA + 0.2 \cdot ML\text{-}NSL)$ according to Panagiotidis and Witt
Sagittal Analysis	SN-Ba	°	Central saddle angle. It describes the extent of the skull base flexion
Sagittal Analysis	SNPg	°	Theangle between Sella, Nasion and Pogonion
Sagittal Analysis	S-N	mm	The S-N line represents the anterior cranial base. It is constructed by connecting the points sella turcica and the Nasion
Sagittal Analysis	Go-Me	mm	The mandibular plane as a line connecting the points gonion and menton
Sagittal Analysis	Wits	mm	measures the extent to which the jaws are related to each other anteroposteriorly.
Growth Analysis	ML-NSL	°	The angle formed between the ML and NSL lines
Dental Analysis	(+1/NL)	°	The angle between upper incisors’ tooth axis and line NL
Dental Analysis	(+1/SN)	°	The angle between upper incisors’ tooth axis and line SN
Dental Analysis	+1/NA	°	The angle between upper incisors’ tooth axis and line NA
Dental Analysis	+1/NA	mm	the upper central incisor to N-A reading in millimeters provides information on the relative forward or backward positioning of the incisor teeth to the N-A line
Dental Analysis	-1/ML (anatomic)	°	The relative anteroposterior angulation of the lower incisor teeth is determined by relating the most protruding incisor tooth to the mandibular plane (ML)
Dental Analysis	(-1/NB)	°	The lower central incisor to N-B reading in degrees indicates the relative axial inclination of these teeth
Dental Analysis	(-1/NB)	mm	The lower incisor to NB line measurement in millimeters shows the relative forward or backward positioning of these teeth to the N-B line
Dental Analysis	Interincisal angle	°	the inter-incisal angle relates the relative position of the upper incisor to that of the lower incisor

Supplementary Table S2.

Parameter	Group A _ Group B	Difference	Lower CI	Upper CI	Adj. P value
NL-ML angle	II_Female-I_Female	-2.17	-4.25	-0.08	0.04
NL-ML angle	II_Male-I_Female	-2.72	-5.23	-0.22	0.03
NL-ML angle	II_14<Age<20-I_0<Age<13	-3.86	-7.13	-0.58	0.01
NL-ML angle	II_14<Age<20-I_Age>21	-3.74	-7.24	-0.23	0.03
NL-ML angle	II_Female_14<Age<20-I_Female_0<Age<13	-5.72	-10.49	-0.95	0.01
NL-NSL angle	II_Female-I_Male	1.41	0.01	2.80	0.05
PFH/AFH	II_Male-I_Female	2.21	0.19	4.24	0.03
Gonial angle	II_Female-I_Female	-4.58	-7.07	-2.09	0.00
Gonial angle	II_Male-I_Female	-4.10	-7.09	-1.10	0.00
Gonial angle	II_Female-I_Male	-3.20	-6.24	-0.15	0.04
Gonial angle	II_14<Age<20-I_0<Age<13	-7.08	-11.00	-3.15	0.00
Gonial angle	II_14<Age<20-I_14<Age<20	-4.77	-7.96	-1.59	0.00
Gonial angle	II_14<Age<20-I_Age>21	-6.02	-10.22	-1.83	0.00
Gonial angle	II_Female_14<Age<20-I_Female_0<Age<13	-8.05	-13.79	-2.31	0.00
Gonial angle	II_Female_14<Age<20-I_Female_14<Age<20	-5.83	-10.40	-1.27	0.00
Gonial angle	II_Female_14<Age<20-I_Female_Age>21	-6.83	-12.41	-1.26	0.00
Gonial angle	II_Female_14<Age<20-I_Male_0<Age<13	-6.85	-13.52	-0.18	0.04
Facial axis	II_Age>21-I_14<Age<20	-2.77	-4.99	-0.55	0.01
Facial axis	II_Female_Age>21-I_Male_0<Age<13	-4.27	-8.46	-0.09	0.04
Facial axis	II_Female_Age>21-I_Male_14<Age<20	-3.46	-6.90	-0.02	0.05
SNB angle	II_Female-I_Female	-1.73	-3.26	-0.19	0.02
SNB angle	II_Male-I_Female	-2.28	-4.13	-0.43	0.01
SNB angle	II_Female-I_Male	-2.42	-4.30	-0.54	0.01
SNB angle	II_Male-I_Male	-2.97	-5.11	-0.83	0.00
SNB angle	II_0<Age<13-I_0<Age<13	-2.85	-5.43	-0.27	0.02
SNB angle	II_0<Age<13-I_14<Age<20	-3.44	-5.59	-1.28	0.00
SNB angle	II_Age>21-I_14<Age<20	-2.83	-5.16	-0.50	0.01
SNB angle	II_0<Age<13-I_Age>21	-3.04	-5.78	-0.30	0.02
SNB angle	II_Male_0<Age<13-I_Female_14<Age<20	-3.88	-7.46	-0.31	0.02
SNB angle	II_Female_Age>21-I_Male_14<Age<20	-3.64	-7.27	-0.01	0.05
SNB angle	II_Male_0<Age<13-I_Male_14<Age<20	-5.01	-8.95	-1.06	0.00
ANB angle	II_Female-I_Female	2.05	1.50	2.60	0.00
ANB angle	II_Male-I_Female	1.83	1.16	2.49	0.00
ANB angle	II_Female-I_Male	2.44	1.77	3.12	0.00
ANB angle	II_Male-I_Male	2.22	1.45	2.99	0.00
ANB angle	II_0<Age<13-I_0<Age<13	1.84	0.90	2.78	0.00
ANB angle	II_14<Age<20-I_0<Age<13	1.95	1.06	2.83	0.00

ANB angle	II_Age>21-I_0<Age<13	2.23	1.24	3.22	0.00
ANB angle	II_0<Age<13-I_14<Age<20	2.03	1.25	2.82	0.00
ANB angle	II_14<Age<20-I_14<Age<20	2.14	1.42	2.86	0.00
ANB angle	II_Age>21-I_14<Age<20	2.42	1.58	3.27	0.00
ANB angle	II_0<Age<13-I_Age>21	2.03	1.03	3.03	0.00
ANB angle	II_14<Age<20-I_Age>21	2.13	1.19	3.08	0.00
ANB angle	II_Age>21-I_Age>21	2.42	1.37	3.47	0.00
ANB angle	II_Female_14<Age<20-I_Female_0<Age<13	1.60	0.31	2.88	0.00
ANB angle	II_Female_Age>21-I_Female_0<Age<13	1.84	0.44	3.25	0.00
ANB angle	II_Female_0<Age<13-I_Female_14<Age<20	2.06	0.90	3.22	0.00
ANB angle	II_Female_14<Age<20-I_Female_14<Age<20	2.29	1.26	3.31	0.00
ANB angle	II_Female_Age>21-I_Female_14<Age<20	2.53	1.37	3.70	0.00
ANB angle	II_Male_0<Age<13-I_Female_14<Age<20	2.11	0.82	3.40	0.00
ANB angle	II_Male_14<Age<20-I_Female_14<Age<20	1.95	0.68	3.22	0.00
ANB angle	II_Male_Age>21-I_Female_14<Age<20	2.27	0.53	4.01	0.00
ANB angle	II_Female_0<Age<13-I_Female_Age>21	1.80	0.43	3.16	0.00
ANB angle	II_Female_14<Age<20-I_Female_Age>21	2.02	0.77	3.27	0.00
ANB angle	II_Female_Age>21-I_Female_Age>21	2.27	0.90	3.64	0.00
ANB angle	II_Male_0<Age<13-I_Female_Age>21	1.85	0.37	3.32	0.00
ANB angle	II_Male_14<Age<20-I_Female_Age>21	1.69	0.23	3.14	0.01
ANB angle	II_Male_Age>21-I_Female_Age>21	2.00	0.12	3.89	0.03
ANB angle	II_Female_0<Age<13-I_Male_0<Age<13	2.48	0.89	4.07	0.00
ANB angle	II_Female_14<Age<20-I_Male_0<Age<13	2.71	1.21	4.20	0.00
ANB angle	II_Female_Age>21-I_Male_0<Age<13	2.95	1.36	4.55	0.00
ANB angle	II_Male_0<Age<13-I_Male_0<Age<13	2.53	0.84	4.22	0.00
ANB angle	II_Male_14<Age<20-I_Male_0<Age<13	2.37	0.70	4.04	0.00
ANB angle	II_Male_Age>21-I_Male_0<Age<13	2.69	0.63	4.74	0.00
ANB angle	II_Female_0<Age<13-I_Male_14<Age<20	1.94	0.63	3.24	0.00
ANB angle	II_Female_14<Age<20-I_Male_14<Age<20	2.16	0.97	3.35	0.00
ANB angle	II_Female_Age>21-I_Male_14<Age<20	2.41	1.10	3.72	0.00
ANB angle	II_Male_0<Age<13-I_Male_14<Age<20	1.98	0.56	3.41	0.00
ANB angle	II_Male_14<Age<20-I_Male_14<Age<20	1.83	0.42	3.23	0.00
ANB angle	II_Male_Age>21-I_Male_14<Age<20	2.14	0.30	3.99	0.01
ANB angle	II_Female_0<Age<13-I_Male_Age>21	2.73	0.58	4.87	0.00
ANB angle	II_Female_14<Age<20-I_Male_Age>21	2.95	0.88	5.02	0.00
ANB angle	II_Female_Age>21-I_Male_Age>21	3.20	1.05	5.35	0.00
ANB angle	II_Male_0<Age<13-I_Male_Age>21	2.77	0.55	4.99	0.00
ANB angle	II_Male_14<Age<20-I_Male_Age>21	2.62	0.41	4.82	0.01
ANB angle	II_Male_Age>21-I_Male_Age>21	2.93	0.43	5.44	0.01
SN-Pg angle	II_Female-I_Female	-1.65	-2.81	-0.48	0.00

SN-Pg angle	II_Male-I_Female	-1.50	-2.91	-0.10	0.03
SN-Pg angle	II_Female-I_Male	-2.48	-3.91	-1.05	0.00
SN-Pg angle	II_Male-I_Male	-2.34	-3.97	-0.71	0.00
SN-Pg angle	II_Age>21-I_0<Age<13	-2.16	-4.22	-0.09	0.04
SN-Pg angle	II_0<Age<13-I_14<Age<20	-2.64	-4.27	-1.00	0.00
SN-Pg angle	II_Age>21-I_14<Age<20	-2.93	-4.70	-1.17	0.00
SN-Pg angle	II_0<Age<13-I_Age>21	-2.59	-4.67	-0.51	0.01
SN-Pg angle	II_Age>21-I_Age>21	-2.88	-5.07	-0.70	0.00
SN-Pg angle	II_Female_0<Age<13-I_Female_14<Age<20	-2.45	-4.87	-0.02	0.05
SN-Pg angle	II_Female_Age>21-I_Female_14<Age<20	-2.69	-5.13	-0.25	0.02
SN-Pg angle	II_Female_0<Age<13-I_Male_14<Age<20	-3.45	-6.19	-0.72	0.00
SN-Pg angle	II_Female_Age>21-I_Male_14<Age<20	-3.70	-6.45	-0.95	0.00
S-N (mm)	II_Male-I_Female	2.96	0.25	5.66	0.03
S-N (mm)	II_Female-I_Male	-2.82	-5.57	-0.07	0.04
S-N (mm)	II_Male_0<Age<13-I_Female_0<Age<13	6.38	0.28	12.47	0.03
S-N (mm)	II_Female_14<Age<20-I_Male_14<Age<20	-5.05	-9.86	-0.24	0.03
Go Me (mm)	II_Female-I_Male	-2.85	-5.24	-0.45	0.01
Wits appraisal	II_Female-I_Female	2.55	1.71	3.39	0.00
Wits appraisal	II_Male-I_Female	2.40	1.39	3.40	0.00
Wits appraisal	II_Female-I_Male	1.55	0.52	2.57	0.00
Wits appraisal	II_Male-I_Male	1.39	0.22	2.56	0.01
Wits appraisal	II_0<Age<13-I_0<Age<13	1.82	0.40	3.25	0.00
Wits appraisal	II_14<Age<20-I_0<Age<13	1.82	0.47	3.17	0.00
Wits appraisal	II_Age>21-I_0<Age<13	2.19	0.68	3.70	0.00
Wits appraisal	II_0<Age<13-I_14<Age<20	2.00	0.81	3.19	0.00
Wits appraisal	II_14<Age<20-I_14<Age<20	2.00	0.91	3.09	0.00
Wits appraisal	II_Age>21-I_14<Age<20	2.36	1.08	3.65	0.00
Wits appraisal	II_0<Age<13-I_Age>21	2.48	0.96	3.99	0.00
Wits appraisal	II_14<Age<20-I_Age>21	2.47	1.03	3.91	0.00
Wits appraisal	II_Age>21-I_Age>21	2.84	1.25	4.43	0.00
Wits appraisal	II_Female_14<Age<20-I_Female_0<Age<13	2.14	0.19	4.09	0.02
Wits appraisal	II_Female_0<Age<13-I_Female_14<Age<20	2.37	0.62	4.13	0.00
Wits appraisal	II_Female_14<Age<20-I_Female_14<Age<20	2.75	1.20	4.30	0.00
Wits appraisal	II_Female_Age>21-I_Female_14<Age<20	2.72	0.96	4.49	0.00
Wits appraisal	II_Male_0<Age<13-I_Female_14<Age<20	2.70	0.74	4.66	0.00
Wits appraisal	II_Male_14<Age<20-I_Female_14<Age<20	1.92	0.00	3.84	0.05
Wits appraisal	II_Male_Age>21-I_Female_14<Age<20	3.35	0.72	5.99	0.00
Wits appraisal	II_Female_0<Age<13-I_Female_Age>21	2.61	0.54	4.67	0.00
Wits appraisal	II_Female_14<Age<20-I_Female_Age>21	2.99	1.09	4.88	0.00
Wits appraisal	II_Female_Age>21-I_Female_Age>21	2.96	0.88	5.03	0.00

Wits appraisal	II_Male_0<Age<13-I_Female_Age>21	2.94	0.70	5.18	0.00
Wits appraisal	II_Male_Age>21-I_Female_Age>21	3.59	0.74	6.44	0.00
+1/NA angle	II_Male-I_Female	-4.26	-8.22	-0.30	0.03
+1/NA angle	II_Male_14<Age<20-I_Female_0<Age<13	-9.09	-17.82	-0.36	0.03
+1/NA angle	II_Male_14<Age<20-I_Female_14<Age<20	-9.07	-16.50	-1.64	0.00
-1/ML (anatomic)	II_Female-I_Female	3.90	1.17	6.63	0.00
-1/ML (anatomic)	II_Male-I_Female	3.58	0.29	6.86	0.03
-1/ML (anatomic)	II_14<Age<20-I_0<Age<13	4.80	0.46	9.13	0.02
-1/ML (anatomic)	II_14<Age<20-I_14<Age<20	3.64	0.12	7.16	0.04
-1/ML (anatomic)	II_14<Age<20-I_Age>21	6.45	1.81	11.08	0.00
-1/ML (anatomic)	II_Female_14<Age<20-I_Female_14<Age<20	5.62	0.64	10.60	0.01
-1/ML (anatomic)	II_Female_14<Age<20-I_Female_Age>21	7.81	1.72	13.89	0.00

Supplementary Figure S1.

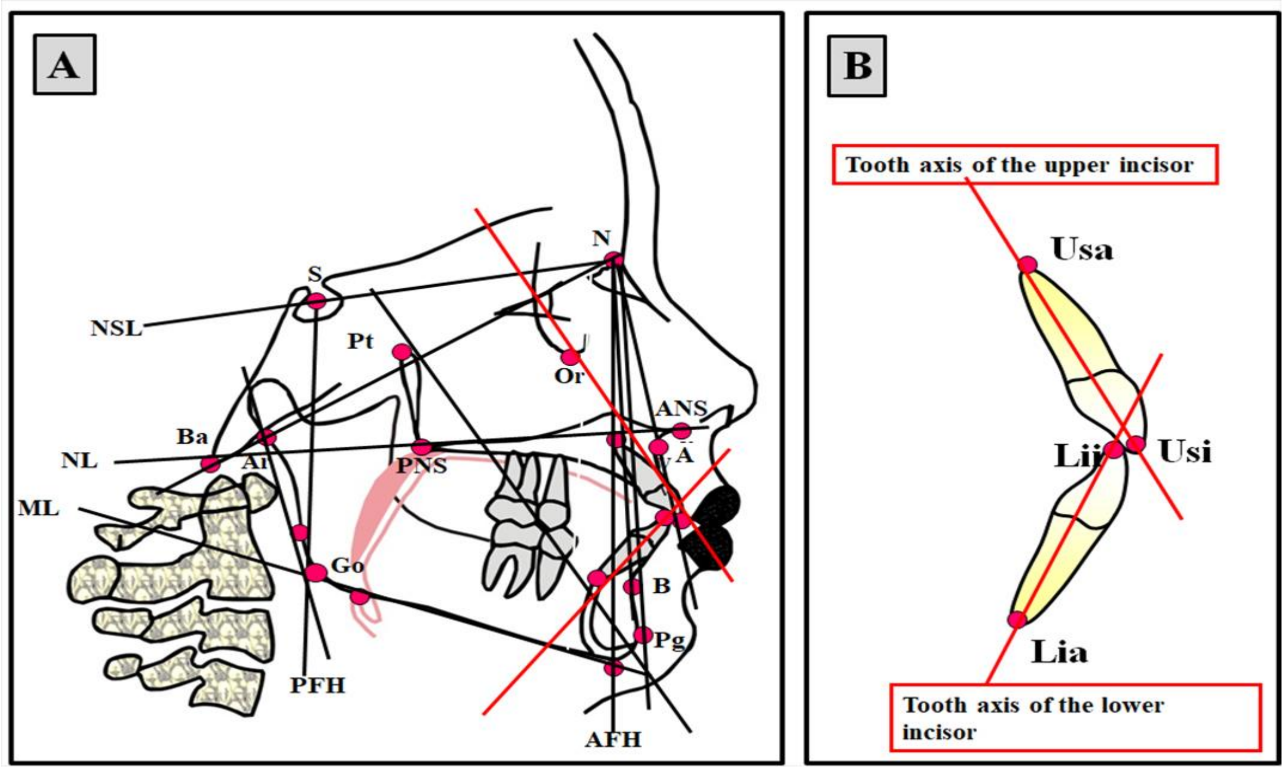

Supplementary Figure S2.

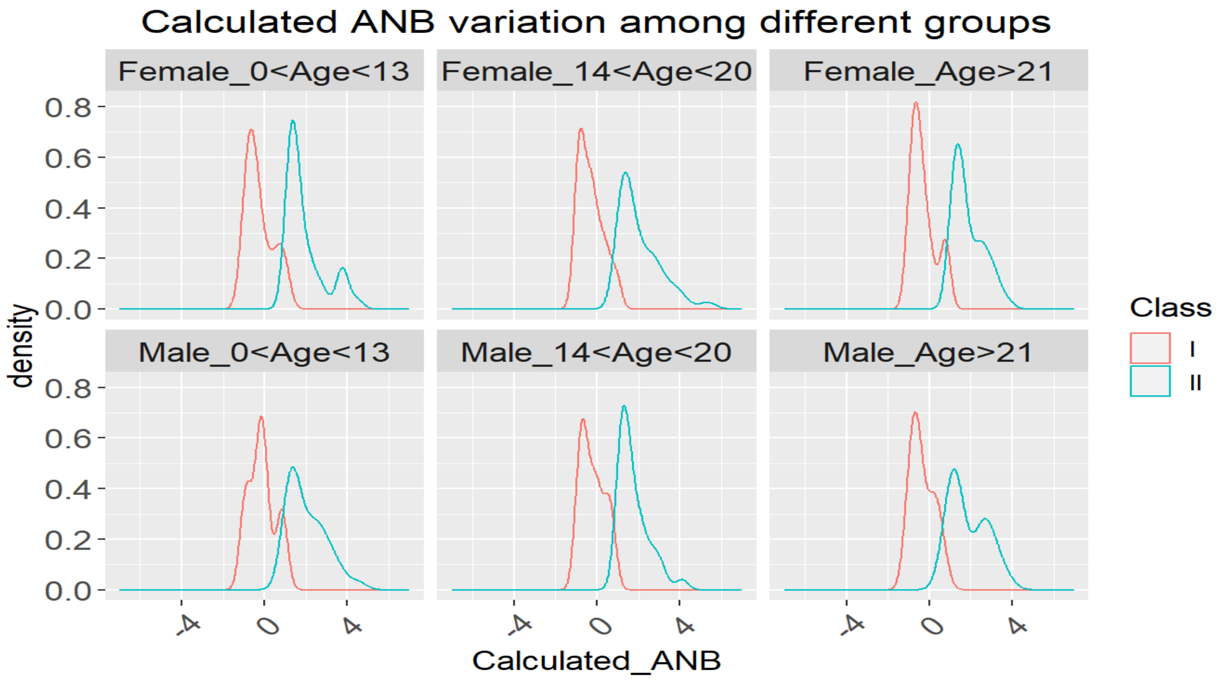

Supplement: Supplementary file 1 [file jcm-14-05292-s001.zip › jcm-3749845-supplementary.pdf]
